# Autoindexing with outlier rejection and identification of superimposed lattices

**DOI:** 10.1107/S0021889810010782

**Published:** 2010-04-30

**Authors:** Nicholas K. Sauter, Billy K. Poon

**Affiliations:** a Physical Biosciences Division, Lawrence Berkeley National Laboratory, One Cyclotron Road, Berkeley, CA 94720, USA

**Keywords:** autoindexing, outlier rejection, superimposed lattices, sample quality

## Abstract

Constructing a model lattice to fit the observed Bragg diffraction pattern is straightforward for perfect samples, but indexing can be challenging when artifacts are present, such as poorly shaped spots, split crystals giving multiple closely aligned lattices and outright superposition of patterns from aggregated microcrystals. To optimize the lattice model against marginal data, refinement can be performed using a subset of the observations from which the poorly fitting spots have been discarded. Outliers are identified by assuming a Gaussian error distribution for the best-fitting spots and points diverging from this distribution are culled. The set of remaining observations produces a superior lattice model, while the rejected observations can be used to identify a second crystal lattice, if one is present. The prevalence of outliers provides a potentially useful measure of sample quality. The described procedures are implemented for macromolecular crystallography within the autoindexing program *labelit.index* (http://cci.lbl.gov/labelit).

## Abstract 2

After autoindexing, Bragg spot candidates that do not fit on the model lattice can be identified, providing a potentially useful measure of sample quality and giving an avenue for indexing a second lattice, if one is present.

## 1. Introduction

**Figure 1:**
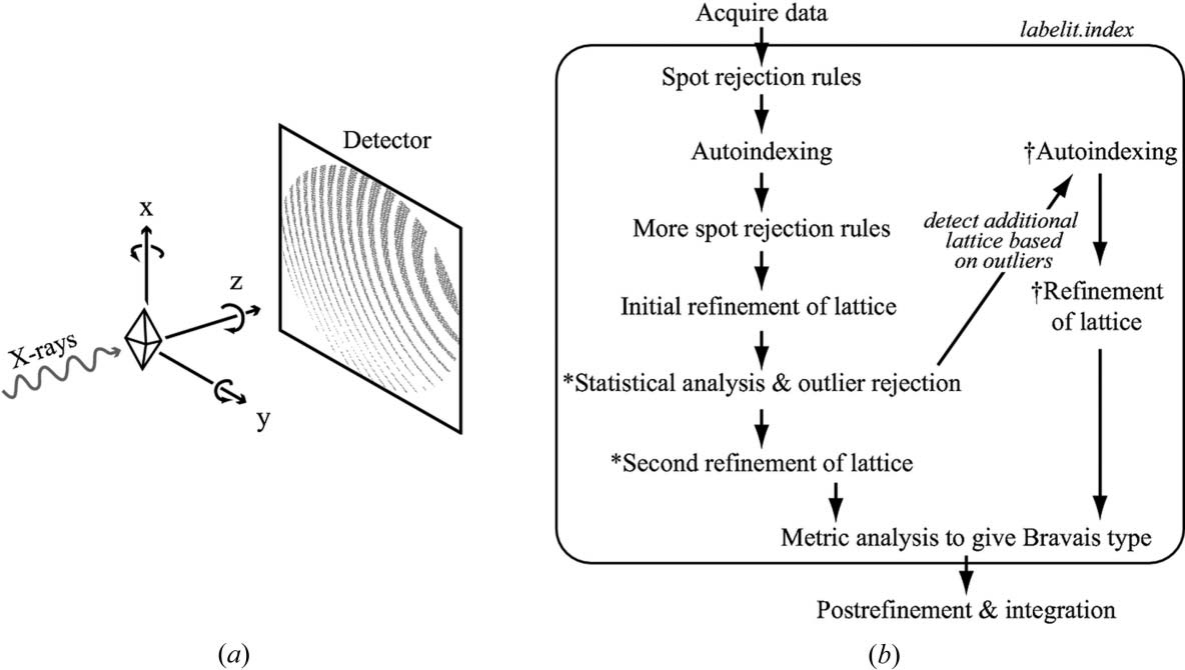
Protocol for outlier detection. (*a*) Data-acquisition geometry, showing librations of the crystal about the incident beam (*z* axis) and goniometer rotation axis (*y* axis). (*b*) Computational procedure showing steps that are executed within the program *labelit.index*. New outlier detection steps developed in this paper are indicated by an asterisk (*) and the alternative pathway for indexing the second lattice (if one is present) is indicated by a dagger symbol (†).

‘How good is this sample?’ and ‘How well does the model fit the data?’ are pertinent questions throughout the process of structure solution, driving critical experimental decisions even at the initial step of eliciting the crystal lattice from the raw diffraction image. Algorithms for determining and refining the lattice description are well understood, and are implemented by many data-processing packages such as *MOSFLM* (Leslie, 1999 ▶), *HKL* (Otwinowski & Minor, 1997 ▶), *XDS* (Kabsch, 2010*a* ▶,*b* ▶) and d*TREK (Pflugrath, 1999 ▶). Generally, candidate Bragg spots are selected from the diffraction image and their observed laboratory coordinates are converted into reciprocal space by an appropriate geometric construction (Arndt & Wonacott, 1977 ▶; reviewed by Dauter, 1999 ▶). Lattice periodicity is detected by one of several autoindexing procedures (*e.g.* Steller *et al.*, 1997 ▶), leading to a lattice model with nine degrees of freedom: three unit-cell lengths, three unit-cell angles and three librations of the lattice with respect to the laboratory axes (Fig. 1 ▶ *a*). The predictive power of the lattice model makes accurate data integration possible; in particular, it is used to deduce image coordinates for all of the reflections (Rossmann & van Beek, 1999 ▶), even those with a low intensity level that would not otherwise be distinguishable from the background level.

The unit-cell model, or rather seven of its nine degrees of freedom (cell lengths, cell angles and libration about the* z* axis or direction of incident X-rays) are readily optimized, since they produce direct changes in the expected Bragg spot positions. By comparing the predicted and observed Bragg positions, a best-fit model can be obtained by least-squares refinement. High-accuracy refinements of librations about the *x* and* y* axes are outside the scope of this paper, as small rotations about these axes do not produce first-order changes in the expected spot positions. The accepted approach in this regard is to integrate the Bragg intensities over a full or partial data set, giving spot-intensity profiles over a sequence of *y* rotations. These profiles determine high-accuracy *x*- and *y*-rotational orientations through a post-refinement algorithm (Rossmann *et al.*, 1979 ▶; Winkler *et al.*, 1979 ▶), and the resulting lattice model is then used as the basis for a second, more accurate, round of integration.

**Figure 2:**
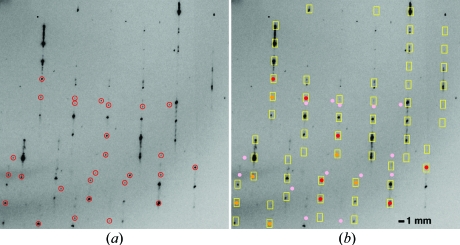
Detail of the 1° rotation image used for analyzing outliers for Protein Data Bank (PDB) entry 1vk8. (*a*) Red circles are the candidate Bragg spots accepted for the initial lattice refinement, after two rounds of heuristic spot filters. Spot intensity is one criterion for accepting these candidate observations, but other characteristics have disqualifed many of the brightest Bragg spots in this case. Specifically, the heuristic rules select signals for which the centroids are extremely well measured, namely round sharp spot profiles that are baseline-separated from neighboring signals. Thus, the Bragg signals exhibiting satellite spots and streaks oriented along the vertical c*-axis direction are excluded from refinement. (*b*) Yellow boxes show the predicted Bragg positions on the initially refined lattice model. Observations have been recolored to indicate their status with respect to this predicted lattice. Red dots represent the 40% of spots closest to their predicted positions, used for determining the Rayleigh distribution σ in Fig. 3 ▶(*c*). Pink spots are those determined to be outliers by the statistical test [equation (6)], very much in agreement with the visual impression. Orange dots are the remaining observations.

This paper focuses only on what is achievable with the observed center-of-mass spot positions from the best-measured spots (Fig. 2 ▶ *a*). There is good reason to emphasize this initial characterization of raw images (prior to data reduction), since high-throughput crystal screening is relied upon increasingly to identify the best crystalline samples prior to the collection of full data sets. Crystal screening, which is now a standard option at many synchroton beamlines (Soltis *et al.*, 2008 ▶), examines numerous samples sequentially under robotic control. A typical protocol involves the collection of two diffraction patterns spaced 90° apart on the *y*-rotational axis, which is enough data to gain a general understanding of the quality of the sample. Software server frameworks such as *Web-Ice* (González *et al.*, 2008 ▶) or *EDNA* (Incardona *et al.*, 2009 ▶) execute autoindexing trials for each crystal, generating a summary report that lists characteristics such as the signal strength and limiting resolution. One important quality measure is the r.m.s. deviation (in laboratory space) between the observed and predicted positions of the best-measured spots. Even in the absence of post-refinement, the lattice model ought to predict spot positions with sub-pixel accuracy, so in the best cases the r.m.s. spot displacement is expected to be less than the CCD pixel size, typically about 100 µm.

In less favorable cases, it is challenging to refine the lattice model in a well behaved manner. If the operative method is to minimize the variance 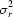 between observed spot centroids **r**_obs_ and predicted spot positions **r**_calc_ for the *N* best-measured spots, 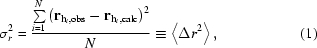
then one must check the implicit assumption that the observations have been paired with the correct Miller index **h**_*i*_. This assumption breaks down for many experimental samples where Miller indices are difficult or impossible to assign. One example is seen in Fig. 2 ▶, where the lattice may be modulated (Wagner & Schönleber, 2009 ▶), generating satellite spots and streaks that are spaced along the c* axis. Another problematic phenomenon (§3) is the superposition of multiple lattices, which requires a decision as to which spots to associate with a given lattice model. Clearly, in these cases, the assignment of observations to the wrong Miller index will artificially inflate the r.m.s. deviation value [which is σ_*r*_ in equation (1)]. Moreover, the refined lattice model will be biased by the outliers, thus degrading any crystal quality measures that depend on an accurate knowledge of the lattice (including the signal strength and limiting resolution). Here we develop a simple statistical test to decide, automatically, which observations should be included in equation (1) and which should be rejected as outliers, thus improving the general computational outcome.

## 2. Computational methods

Raw diffraction images for a number of published protein structures were downloaded from the Joint Center for Structural Genomics (JCSG; http://www.jcsg.org) to be used as test cases. Images from hen egg-white lysozyme containing superimposed lattices from two crystals were obtained by Peter Zwart at Beamline 5.0.1 of the Advanced Light Source at Berkeley. Software development was greatly facilitated by the framework provided by the open-source Computational Crystallography Toolbox (*cctbx*; Grosse-Kunstleve *et al.*, 2002 ▶).

Raw data are analyzed with a spot-finding program (*DISTL*; Zhang *et al.*, 2006 ▶), with a view to eliminating various types of signal artifacts prior to any further analysis. The rules applied (determined by trial and error) have been described elsewhere (Sauter *et al.*, 2004 ▶; Sauter & Zwart, 2009 ▶) and are only briefly repeated here. Spots are retained for analysis only if they have smooth profiles that are well separated from their neighbors. Falloff of the spot count as a function of resolution is used to determine conservative outer- and inner-resolution cutoffs. Additional filters reject spots that are unusually intense, large or small in pixel area, or skewed in shape. Spots are ignored if they are too close to the rotation spindle for accurate positional evaluation.

Further analysis is performed on the spots once the tentative lattice model is established by autoindexing. A formula given previously [equation (8) of Sauter *et al.* (2004 ▶)] converts an observed spot position **r** to a real-valued Miller index **f**, the components of which are rounded off to produce the likely integer-valued Miller index **h**. Index **h** is not always well defined for a rotation photograph; the rotation of the sample about the *y* axis (typically anywhere from 0.1 to 1.0°) may produce differing **h** values for a particular **r** position at the beginning and end of the rotation, in which case the observed spot is removed from consideration. The difference **f** − **h** is expected to have small fractional components. Conversely, large component values may indicate an outlier. Indeed, we are able to filter out ice-ring artifacts by detecting peaks in a plot of |**f** − **h**| *versus* resolution, usefully supplementing the detection of ice rings by additional plots of background-corrected pixel intensities and number of candidate spots *versus* resolution. Yet despite all this attention to heuristics for classifying outliers, there are still numerous examples of undetected split spots, fragmented ice rings and superimposed lattices that work their way into the target function [equation (1)] for optimizing the lattice model.

**Figure 3:**
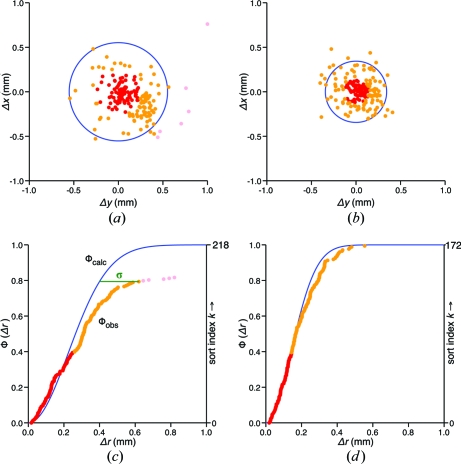
Analysis of 1vk8 outliers. (*a*) and (*b*) are scatter plots of the deviation between observed and predicted Bragg spot positions for (*a*) the initially refined lattice model and (*b*) the re-refined lattice model after outlier rejection. The blue circles in (*a*) and (*b*) are the expected limits that should contain 95% of the spots, based on the Rayleigh distributions modeled in (*c*) and (*d*), respectively. (*c*) and (*d*) are cumulative distribution functions (blue curves) calculated on the subset of observations (red dots) containing the 40% of spots with the smallest values of Δ*r*. Pink dots represent outliers with Δ*r* values more than σ away from the expected curve (green bar). The outliers have been removed in the second refinement round shown in (*b*) and (*d*). Orange dots are the remaining observations.

We therefore resort to statistical assumptions to help sort the spots. Two populations are posited, a collection of outliers that do not fit the lattice model, and a normally distributed collection of lattice spots that fit the model, albeit with some uncertainty. The normally distributed lattice spots are taken to deviate from their predicted positions in the plane of the detector with a Gaussian probability distribution in each coordinate, *x* and *y*. We make the further simplifying assumptions that the individual coordinate deviations Δ*x* and Δ*y* (Fig. 3 ▶ *a*) are uncorrelated and that their variances are equal (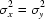). This permits the total deviation between the observed and predicted positions, 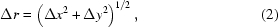
to be modeled with a Rayleigh probability distribution (which may be thought of as an extension of the Gaussian distribution into two dimensions), 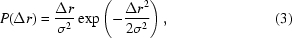
where σ^2^ = 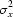 = 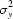 = 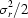.

With this framework in place, the goal now is to separate observations that do and do not appear to fit a Rayleigh distribution. The total group of *N* observations is sorted in order of increasing Δ*r* (Fig. 3 ▶ *c*), indexed by the symbol *k* (*k* = 0, 1, …, *N* − 1). A useful tool for interpreting this series is the cumulative distribution function, or probability that an observation will have a Δ*r* lower than a given value, 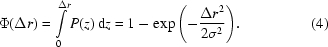
It is assumed that the spots with the smallest Δ*r* values will form a safe group (with no outliers) from which to model the variance in this equation. We therefore take a reasonable subset (40% of the well measured spots with the lowest Δ*r*; see below) and optimize the value of σ by least-squares minimization of a function *f* that characterizes the vertical difference between the observed and calculated cumulative distribution curves in Fig. 3 ▶(*c*), 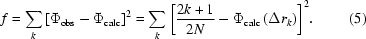
In this equation, the observed distribution function Φ_obs_ is just a straight-line function, (2*k* + 1)/2*N*, that spreads the observations along the vertical axis of Fig. 3 ▶(*c*), while the calculated distribution function Φ_calc_ is equation (4) evaluated at the Δ*r* position of the *k*th observation. Modeling the variance with equation (5) is expected to be superior to using equation (1), since we are fairly confident that equation (5) samples only the variation of spots that truly belong to the lattice, and not the outliers that may be present in the total ensemble of *N* spots. With the cumulative distribution function [equation (4)] now established, it is possible to define an outlier as an observation for which Δ*r* is too far away from the idealized curve of equation (4). A good cutoff is a distance of σ [represented by the green bar in Fig. 3 ▶(*c*)], *i.e.* spots are classified as outliers when 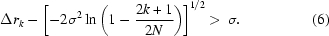
It is important to understand that this cutoff criterion does not penalize observations that depart from the mean by many standard deviations. For example, if the sample size is extremely large it would be perfectly acceptable for a non-outlying spot to have Δ*r* = 6σ. Rather than impose an arbitrary σ cutoff, outliers are identified when the incidence of high Δ*r* values exceeds that expected from a Rayleigh distribution. This approach accounts for the sample size in a natural way, as others have done in different contexts (Read, 1999 ▶; Zwart *et al.*, 2005 ▶).

The statistical model for outlier rejection is applied immediately after autoindexing at the computational stage (Fig. 1 ▶ *b*) where the lattice model is still expressed as a triclinic cell. Subsequent to outlier rejection, the model is re-refined [again based on the target function in equation (1)] and the cell is analyzed to discover the possible Bravais types (Sauter *et al.*, 2006 ▶) prior to data integration.

## 3. Results

**Figure 4:**
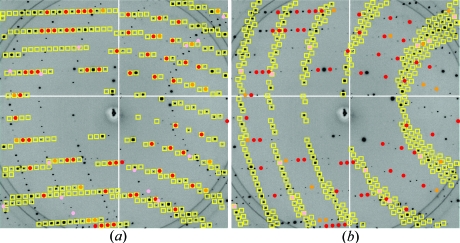
Indexing of two superimposed lysozyme diffraction patterns. (*a*) The primary lattice model (yellow boxes) after an initial round of refinement, where colored dots represent the spots included in the lattice refinement. Color coding is the same as used in Figs. 2 ▶ and 3 ▶. Red spots are the subset safely considered to be part of the primary lattice, while pink spots are the outliers identified by equation (6) (consistent with the visual impression). (*b*) The lattice that results from a second round of autoindexing using only the pink-coded outliers as data. Spot color codes are the same as in (*a*). The new lattice in (*b*) is clearly consistent with the pink (outlying) observations.

To assess the lattice quality from a variety of crystal samples, 22 protein structures were selected from the JCSG data archive, spanning a wide subset of Bravais types. As indicated in Table 1 ▶, diffraction properties such as limiting resolution varied over a considerable range, as did the experimental conditions such as the width of the rotation angle (not shown). Lattice deviation statistics computed from one rotation image in each data set (generally the first image) reveal a broad spectrum of sample qualities, with one sample (3bgu) exhibiting a second lattice and two others presenting Bragg spots that are split in half (1vr8) or streaked in long rows (1vk8; Fig. 2 ▶). Separately, a test image from a lysozyme sample containing two lattices was analyzed (Fig. 4 ▶).

Each diffraction pattern was autoindexed with *LABELIT* (Sauter *et al.*, 2004 ▶) and the resulting lattice refined based on its agreement with the center-of-mass positions of the *N* best-candidate Bragg spots [equation (1)]. Our procedure for rejecting outliers is illustrated in Fig. 3 ▶ for the 1vk8 diffraction pattern. Fig. 3 ▶(*a*) plots the Δ*x* and Δ*y* deviations of the observed spots from their predicted positions, although the outliers that are several millimeters away from the nearest expected lattice point (Fig. 2 ▶ *b*) are beyond the extent of this graph. The ordered sequence of Δ*r* values is shown in Fig. 3 ▶(*c*), along with the best-fit cumulative distribution function [Φ(Δ*r*), blue curve] which is modeled on the 40% of observations with the lowest Δ*r* values (red spots). In all cases examined, choosing the best-conforming subset of Bragg spots in this manner shows that the errors in the spot positions are described reasonably well by a Rayleigh distribution [equations (3) and (4)]. The modeled parameter describing the distribution (σ) is not very sensitive to the exact fraction of spots included; comparable results are obtained upon inclusion of anywhere from 25 to 55% of the spots. A command-line option was added to *labelit.index* to set this parameter (see §4).

In the case of sample 1vk8, a full 80% of the chosen spots coincide with the Rayleigh distribution (blue line, Fig. 3 ▶ *c*), while the remaining observations (those above the green cutoff line) depart markedly from this ideal. These are interpreted as outliers, and are thus removed from the list of observations. Another round of lattice refinement ensues, this time producing Δ*x* and Δ*y* deviations with a much tighter distribution about the model (Fig. 3 ▶ *b*). Also, an ordered plot of Δ*r* values shows that the entire set of remaining observations adheres extremely well to a Rayleigh distribution (Fig. 3 ▶ *d*). The improvement in the model is not confined to the 1vk8 case; indeed, the r.m.s. deviation between model and experiment (σ_*r*_) decreases after a second round of model refinement, sometimes dramatically, in every instance where outliers are rejected (Table 1 ▶).

For the crystals containing two lattices (3bgu and lysozyme), the initial lattice refinement target (for refining the predominant lattice) is contaminated with spot observations from the second lattice. The r.m.s. spot deviations (887 and 586 µm, respectively) are therefore exceptionally large. However, the statistical rejection of outliers successfully removes these observations, such that the second round of lattice refinement produces much lower spot deviations (66 and 100 µm) that are typical of single-crystal samples. Furthermore, the identification of the outlying spots provides an opportunity to index the second lattice: a separate round of autoindexing based on just the outliers (Fig. 1 ▶ *b*) produces good lattice models, although the spot deviations of 586 and 321 µm are fairly high. The significance of this result is that it is not necessary to determine manually which spots to identify with the second lattice prior to autoindexing based on painstaking visual inspection. The two superimposed lysozyme lattices discerned in this manner are depicted in Fig. 4 ▶.

As explained in §1 above, the automated crystal-screening experiments that are the intended focus of this paper often rely on acquiring two images spaced 90° apart on the *y*-rotational axis. We simulated such data sets by selecting widely spaced images from the JCSG archive. Statistical rejection of outliers from these two-image data sets (data not shown) did not differ remarkably from the one-image trials listed in Table 1 ▶, either in the ability to improve the model fit or the ability to conform the remaining spots to a Rayleigh model. Certain minor details did change when considering the two images together, *e.g.* the additional lattice was not as pronounced in the second 3bgu image and the spot splitting was not as severe in the second 1vr8 image. In other data sets such as 1vm6, the percentage of spots rejected as outliers increased slightly, apparently because the lattice model proved to be a better fit for one of the two images (data not shown).

## 4. Discussion

Within the crystal-screening paradigm, tens or hundreds of similar crystal samples may be evaluated for optimal diffraction before one or two are selected for data collection (Page *et al.*, 2005 ▶). Automated software tools can facilitate this process by providing measures of sample quality (such as the signal-to-noise ratio, limiting resolution, r.m.s. deviation, mosaicity and number of ice-ring artifacts) in real time as the data are acquired. Except for the quantification of ice rings, these standard quality measures focus on the diffracted lattice itself. What have been lacking are reliable measures for the interference caused by non-lattice artifacts, which might degrade the integration of the Bragg signal or the subtraction of background, thus reducing the quality of the structure factors. Two of the statistics presented in Table 1 ▶ appear to capture this information. Firstly, the number of outliers (expressed as a percentage of total Bragg spot candidates after ice rings have been removed) measures the prevalence of signals that do not belong to the canonical lattice. Secondly, the severity of the outliers (computed as the Fig. 3 ▶ *c* area bounded by the observed spots, the blue curve and the green cutoff line, in units of σ) gauges how far the outliers are from the lattice. High values of these measures in Table 1 ▶ correlate with the visual recognition of stray spots in the 1vk8, 3bgu, 1vr8 and lysozyme images. These statistics may therefore prove useful for crystal screening in combination with the standard measures mentioned above.

The new methods described here – statistical rejection of outliers followed by re-refinement of the lattice model – have been added to the default flowchart within the autoindexing program *labelit.index* (Fig. 1 ▶ *b*). Although the program normally operates with image data as the only input, a few command-line options have been added (described in the online manual) for generating plots such as those shown in Figs. 2 ▶–4 ▶ ▶, or for disabling outlier rejection altogether. Presently, the indexing of a second lattice is not part of the default procedure. Rather, it is intended that a high percentage of outliers will alert the user to the possibility of a second lattice, whereupon *labelit.index* may be run a second time with a command-line flag set to follow the ‘additional lattice’ indexing path shown in Fig. 1 ▶(*b*).

The discovery of additional lattices performed here and elsewhere (Buts *et al.*, 2004 ▶) raises the question of how to handle data reduction. Standard programs such as *MOSFLM*, *HKL*, *XDS* and d*TREK treat only one lattice at a time, so separate data-reduction runs will have to be performed to integrate the Bragg signals from each lattice. Care must be taken with pairs of reflections from different lattices that either overlap or come close enough such that the background subtraction performed for one lattice is biased by the Bragg signal from another lattice. One approach, implemented in the program *UNTANGLE* (Buts *et al.*, 2004 ▶), is to enumerate and reject problematic pairs of reflections before the data sets are merged together. Other programs such as *PROW* (Bourgeois *et al.*, 1998 ▶) and *EVAL15* (Schreurs *et al.*, 2010 ▶) preserve the information that is present in near or overlapping signals by jointly integrating and deconvoluting the neighboring spots.

The need for software tools to analyze multiple lattices is likely to increase. In contrast with the present practice of transferring individual crystals to the goniometer stage, some new sample-preparation technologies have focused on the *in situ* collection of data without removing crystals from their growth chamber. These protocols, involving samples grown in capillaries (Yadav *et al.*, 2005 ▶) or on microfluidic plates (Ng *et al.*, 2008 ▶; Gerdts *et al.*, 2008 ▶; Emamzadah *et al.*, 2009 ▶), omit the step where crystals are separated from each other, so it is quite possible for the incident X-ray beam to impinge on two crystals simultaneously. The early outlier-based detection of additional lattices (§3) could either be used automatically to trigger special data-reduction protocols, or be deployed as part of a system of safeguards to avoid collecting such data sets altogether.

At the same time, one must keep in mind the assumptions underlying the present methods. Autoindexing must succeed prior to outlier analysis, so even if there are multiple lattices, one of them must be sufficiently predominant so that its unit-cell axes can be discerned by the autoindexing methodology (Steller *et al.*, 1997 ▶, in the case of *labelit.index*). Outlier detection relies on the assumption that there is a safely known subset of observations (we assume 40% here) that truly coincides with the main lattice. Finally, if the outliers are used as a basis for determining a second lattice, we again assume that there is a predominant signal to be found, so it may be difficult to apply the present method to a superposition of three or more lattices.

The procedures described here are included in the software package *LABELIT*, available for download by non-commercial users at http://cci.lbl.gov/labelit, and for licensing by commercial users. *LABELIT* is also included with the *PHENIX* package (Adams *et al.*, 2002 ▶, 2010 ▶), available for download at http://www.phenix-online.org.

## Figures and Tables

**Table 1:** Improvement of the lattice model after outlier rejection Results shown here represent the analysis of a single rotation image. σ_*r*_ is the r.m.s. difference between observed and predicted spot positions as defined in equation (1). σ is a standard deviation fitted to the 40% of observations (before outlier rejection) that are closest to their corresponding predicted positions, as illustrated in Fig. 3 ▶(*c*).

PDB code	Published space group	Limiting resolution (Å)	*N*	σ_*r*_ before outlier rejection (µm)	σ_*r*_ after outlier rejection (µm)	σ before outlier rejection (µm)	Number of outliers (%)	Severity of outliers (σ)	σ_*r*_ on the second lattice (µm)
1vkk	*P*1	1.4	205	388	102	73	7.8	15.7	
1zcz	*P*1	2.1	272	268	103	85	2.6	10.2	
1vk8	*P*1	2.1	218	927	220	225	20.2	81.8	Streaky spots
2rh0	*P*1	3.2	95	529	206	198	9.5	12.7	
3bgu	*P*2	1.8	207	887	66	89	34.3	85.7	586
1vkh	*P*2_1_	1.9	280	266	115	82	7.5	14.0	
1vm6	*C*2	3.1	247	338	56	56	8.5	17.0	
1vl7	*P*2_1_2_1_2	1.9	278	76	72	46	0.7	0.5	
1vph	*P*2_1_2_1_2_1_	2.0	357	221	113	84	7.0	12.9	
1vl1	*C*222_1_	2.0	260	118	57	41	18.5	10.3	
1z9f	*F*222	2.7	137	131	131	80	0.0	0.0	
1vky	*I*222	2.5	273	136	124	83	2.6	2.5	
3b77	*P*4	3.2	296	61	29	22	16.6	5.8	
1vrd	*I*4	3.6	231	149	91	68	16.0	10.7	
1o3u	*P*4_1_2_1_2	2.0	290	150	91	73	1.7	3.8	
2ax3	*I*422	2.8	274	104	97	71	0.4	0.3	
1vr7	*R*3: *H*	1.6	290	150	82	63	3.1	5.8	
1vr8	*P*3_2_21	2.1	246	362	109	85	23.6	33.3	Split spots
2pfx	*P*6_3_	1.8	459	117	71	56	5.2	7.8	
2r6v	*P*6_1_22	1.5	299	116	74	59	3.0	3.8	
1vmd	*I*23	2.9	266	140	121	80	2.3	2.5	
1vlv	*F*432	2.7	362	56	48	33	3.3	1.8	
Lysozyme	*P*4_3_2_1_2	1.9	175	586	100	119	30.3	49.3	321
